# Comparing qualitative thematic analysis and machine-based topic modelling in the analysis of autistic and ADHD young people’s accounts of emotions

**DOI:** 10.1038/s41598-025-34570-7

**Published:** 2026-01-28

**Authors:** Steve Lukito, Lifang Li, Susie Chandler, Myrofora Kakoulidou, Georgia Pavlopoulou, Maciej Matejko, Isabel Jackson, Beta Balwani, Tiegan Boyens, Dorian Poulton, Luke Harvey-Nguyen, Amber Johnson, Daniel Stahl, Angus Roberts, Edmund J. S. Sonuga-Barke, the RE-STAR Team, Steve Lukito, Maciej Matejko, Beta Balwani, Tiegan Boyens, Dorian Poulton, Luke Harvey-Nguyen, Amber Johnson, Daniel Stahl, Angus Roberts, Edmund Sonuga-Barke, Susie Chandler, Andrea Danese, Johnny Downs, Eloise Funnell, Kirsty Griffiths, Myrofora Kakoulidou, Lauren Low, Umaya Prasad, Emily Simonoff, Anna Wyatt, Georgia Pavlopoulou, Jane Hurry, Sylvan Baker, Graham Moore, Dennis Ougrin, Amanda Roestorf, Rebecca Kirkbride, Jordan Altimimi, Saskia Barnes, Zoë Glen, C. J. Harris, Charlotte Hillman, Issy Jackson, Elisa Ly, Elizabeth Macauley, Anya Rose, Darren Webb, Archie Wilson

**Affiliations:** 1 Department of Child and Adolescent Psychiatry, Institute of Psychiatry, Psychology and Neuroscience, King’s College London, DeCrespigny Park, London, SE5 8AF UK; 2 Department of Biostatistics and Health Informatics, Institute of Psychiatry, Psychology and Neuroscience, King’s College London, London, UK; 3 School of Journalism and Communication, Sun Yat-Sen University, Guangzhou, Guangdong People’s Republic of China; 4 Group for Research in Relationships in Neurodiversity (GRRAND), Institute of Education, Department of Psychology and Human Development, University College London, London, UK; 5 Research Department of Primary Care and Population Health, Faculty of Population Health Sciences, University College London, London, UK; 6 Anna Freud National Centre for Children and Families, London, UK; 7 Psychology and Human Development, Institute of Education, University College London, London, UK; 8 Royal Central School of Speech and Drama, London, UK; 9 Cardiff University, Cardiff, UK; 10 Queen Mary University of London, London, UK; 11 Autistica, London, UK; 12 Place2Be, London, UK

**Keywords:** Autism, ADHD, Reflexive thematic analysis, Topic modelling, Emotional regulation, Neuroscience, Psychology, Psychology

## Abstract

Systematic analysis of interview data can provide important insights into how young people experience and interpret their emotions. Both human-led qualitative (e.g., thematic analysis) and machine-driven quantitative (e.g., natural language processing [NLP]) analytical approaches are available, but their solutions are rarely compared. Interview responses by 57 neurodivergent adolescents to questions about their emotions, previously analysed using reflexive thematic analysis (RTA), were submitted to Topic Modelling (TM). Topic labels were developed in collaboration with neurodivergent co-researchers to ground their meaning in the lived experience of neurodivergent communities. Topics were mapped to RTA themes or subthemes to examine their proximities. Topic-based cluster analysis was used to identify participant groupings with similar topic distributions. TM revealed 10 interpretable and meaningful emotion-related topics – some closely overlapping with and others differing from the RTA themes. TM topics differentiated the young people’s emotional experiences at school from those in other settings. TM and RTA resulted in overlapping and different insights into the meaning of neurodivergent young people’s accounts of their emotions. Our findings demonstrate the potential use of TM in interview analysis and might suggest a potential complementarity between the TM topics and RTA themes, to be further explored using more advanced algorithms and a more sophisticated NLP implementation.

**Supplementary Information:**

The online version contains supplementary material available at 10.1038/s41598-025-34570-7.

## Introduction

Neurodivergent young people who are autistic and/or have attention-deficit/hyperactivity disorder (ADHD) are at an increased likelihood of mental health difficulties, compared to neurotypical peers^1–4^. Among these groups, the experience of strong negative emotions and emotional responses^5–7^ are common and have been shown to mediate the emergence of depression [^8–10^, e.g.,^11^]. Traditionally, emotional responses in neurodivergent young people are reported by adult observers (parent/carer) using measures typically validated first in neurotypical populations^12^.

The RE-STAR (Regulating Emotion – Strengthening Adolescent Resilience) research programme has taken a participatory research approach and placed the neurodivergent young people’s experiences at the heart of its inquiry. We interviewed autistic secondary mainstream school pupils and/or those with ADHD about their emotional lives using a semi-structured interview schedule co-produced with neurodivergent young people. We first explored the young people’s insights into their emotions by using reflexive thematic analysis (RTA)^13^. RTA is an approach to qualitative analysis where researchers actively construct patterns of meaning, or themes, within texts, while emphasising self-awareness and critical reflection, and acknowledging their positionality^14^, which can play multiple important roles in translational research^15^. Our RTA of the above young people’s interview scripts revealed themes relating to: (1) the nature of upsetting events that bring negative emotions in young people, and (2) the sorts of activities or supports that help the young people manage these emotions^16,17^. These provided a basis for the development of a new self-report measure of emotional burden associated with school-based upsetting events^18^.

In this paper, we contrast this with a quantitative machine-learning approach called topic modelling (TM). TM is a statistical technique used to identify hidden themes or ‘topics’ in large collections of text. It works by analysing vocabulary and topic distributions across documents, typically in a large collection^19,20^, allowing the algorithm to group related terms and to assign separate topics to each document. TM is one of many techniques used in natural language processing (NLP) – the computational analysis and representation of natural language text for various applications^21^. Machine learning and modern NLP are data-driven, deriving outputs from the empirical evidence in the textual input, rather than from a theoretical understanding of the text. TM uses an unsupervised machine learning technique, in that it applies an algorithm to generate a statistical model of text from previously unseen data, without the benefit of training, prior data labelling, or supervision from human-labelled examples of similar textual data.

Unlike primarily human-led qualitative analysis, which can be a prolonged and iterative process, especially during the coding stages leading to the derivation of themes, TM can be applied to large datasets with relative ease, forming topics with a high degree of replicability, which can then be interpreted and labelled by humans to ensure that they are contextually meaningful^22^. Despite these strengths, TM does have the disadvantage of requiring large quantities of text for optimal performance, e.g., those derived from discourses in online forums or social media [e.g.,^23–25^], but its potential use in conjunction with qualitative research, typically conducted with fewer participants, has been proposed recently [see e.g.,^26–28^]. Notably, drawing a degree of equivalence between topics and themes, Gillies and colleagues^26^ outlined several possible workflows for combining TM and qualitative research, including, among others, the use of TM topics as a seed or starting point for thematic analysis or the use of qualitative themes derived from a subset of data as a seed for running a subsequent topic model on the remaining data^26^.

While TM’s potential in qualitative enquiry involving a high volume of free-text qualitative datasets (e.g., > 500 participants), too large for a qualitative researcher to manage, has been demonstrated^23,24^, this is less obvious in studies involving verbal interaction like interviews, which typically have considerably fewer participants, e.g., between a minimum of 12 and as many as 50–60 individuals^29–31^. Only a few TM studies involving this kind of data currently exist in the literature. For instance, Bastiaansen and colleagues^32^ compared qualitative themes with TM topics from unstructured interviews with 13 young people about their experiences as siblings of neurodivergent adolescents, while Miyaoka and colleagues^33^ compared themes generated from a ‘pure’ and TM-augmented thematic analysis on focus group conversations of 16 teachers about students’ experience using maths technology. Both found overlaps between qualitative themes and topics or themes generated or augmented by TM, as well as unique output from the respective analyses, although outputs from TM were typically less nuanced than the human-generated themes. Both studies involved data derived from a relatively small sample. Similar exploration involving a larger sample size, where qualitative analysis is more effortful but leads to greater thematic nuances and diversity, could serve as a useful contrast. The larger participant sample size also enables more sophisticated quantitative explorations than in previous studies^32,33^.

Considering the potential overlap and unique contributions from each methodology, we designed a study to investigate the similarities and differences between the TM topics and reflexive themes following the RTA investigation. Using responses from interviews with 57 autistic young people and/or those with ADHD, we conducted an exploratory study investigating similarities or differences between the TM and RTA approaches. We asked: (i) if we could extract meaningful topics from the interview transcripts from these young people using TM; (ii) whether these topics could be relevant clinically, for instance, whether they are associated with the young people’s diagnosis or contextual background, and (iii) how the TM findings compared with the conventional RTA methods. We first applied TM to the young people’s responses to interview questions to generate topics and inspect their contents for clinical relevance. We further investigated whether the distribution of such topics among participants clustered them in a way that was associated with their diagnosis and other characteristics. Finally, we examined how these topics relate, or map, to the RTA themes and subthemes.

## Methods

### Study design

This study utilised TM in secondary analyses of semi-structured interviews from *“My Emotions and Me”* study, which explored the emotional experiences of autistic young people and/or those with ADHD^16^. Interview schedules were co-produced with autistic young adults and/or those with ADHD aged 18–25 years, members of RE-STAR Youth Researcher Panel (Y-RP)^34^ and were administered in school-aged students to explore: (1) what provokes negative emotions in everyday life, and (2) what helps young people to manage the emotions. Each interview took place online with an experienced post-doctoral level researcher (MK/SL) for approximately 90 minutes. The study received ethics approval from UK National Health Service Research Ethics Committee (22/NI/0017) and was conducted in accordance with the Declaration of Helsinki. Informed written parental consent and young person assent were obtained for all participants. For a detailed account of the interview approach and main qualitative findings, see Pavlopoulou^16^.

### Participants

Participants were 57 mainstream secondary school students aged 11 to 15 years (*M*_age_ ± *SD* = 13.0 ± 1.36 years; 19 female) and with sufficient use and understanding of spoken English to complete the interviews. Nine participants (15.8%) were on free school meals, 14 (24.6%) were non-white. Twenty-one had a diagnosis of autism, 24 had ADHD, and 12 had autism + ADHD. Thirty-two (56.1%) received at least one medication. Psychostimulants were the most common, taken by 20 (35.1%) participants with ADHD or with autism + ADHD (Table 1). The participants were recruited through autism and ADHD charities, social media, and the South London and Maudsley NHS Foundation Trust.

**Table 1:** Participant characteristics.

	Overall (n = 57)	Autism (n = 21)	ADHD (n = 24)	Autism + ADHD (n = 12)
Age (M, SD), years	13.0 (1.36)	12.9 (1.35)	13.0 (1.40)	13.2 (1.40)
Female (n, %)	19 (33.3)	10 (47.6)	6 (25.0)	3 (25.0)
On free school meals (n, %)	9 (15.8)	2 (9.5)	5 (20.8)	2 (16.7)
Ethnicity (n, %)				
White	43 (75.4)	16 (76.2)	19 (79.2)	8 (66.7)
Black African/Caribbean/British	4 (7.0)	1 (4.8)	1 (4.2)	2 (16.7)
Asian/Asian British	3 (5.3)	0 (0.0)	1 (4.2)	2 (16.7)
Mixed/multiple ethnic backgrounds	5 (8.8)	3 (14.3)	2 (8.3)	0 (0.0)
Other ethnicities	2 (3.5)	1 (4.8)	1 (4.2)	0 (0.0)
Medication (n, %)				
Psychostimulants	20 (35.1)	0 (0.0)	14 (58.3)	6 (50.0)
Sleep medication	13 (22.8)	5 (23.8)	5 (20.8)	3 (25.0)
SSRIs	2 (3.5)	2 (9.5)	0 (0.0)	0 (0.0)
Asthma medication	3 (5.3)	2 (9.5)	0 (0.0)	1 (8.3)

ADHD = attention-deficit/hyperactivity disorder, M = mean, SD = standard deviation, n = number of participants, % = percentage in the sample, SSRIs = selective serotonin reuptake inhibitors.

### Reflexive thematic analyses

The RTA was fully described in a separate publication^16^. Briefly, individual interview accounts were transcribed and analysed using NVivo 1.6.1 (QSR International Pty Ltd). The analysis steps consisted of researchers’ familiarisation with the interview transcripts, development of codes related to the research questions, and the generation of interview subthemes and themes, along with their nuances based on Y-RP input^13^. These were further checked by a subset of participants, reflecting our commitment to participatory rights-based research^35^.

RTA yielded shared themes across the diagnostic groups (i.e., autism, ADHD, autism + ADHD) around everyday upsetting experiences including: (1) ‘under-/over-stimulation and sensory mismatch’, consisting of the subthemes ‘over-stimulated’ and ‘under-stimulated’; (2) ‘social dislocation, alienation and conflict’, consisting of the subthemes of ‘stigma, conflict and victimisation’ and ‘a mismatch of salience’, i.e., the mismatching of expectations or perceptions of events between others and the young person; (3) ‘experience of hiding one’s true self, the need to mask’, consisting of the subthemes ‘efforts to protect others from my feelings’ and ‘a constant need to protect self from others’ reactions’; and (4) ‘self-doubt, loathing, embarrassment’, consisting of the subthemes ‘being invisible and dismissed’, and ‘guilt and embarrassment when help is needed’^16^. Furthermore, responses to the question of what helps to manage negative emotions revealed three broad themes: (5) ‘what helps prevent experiences from becoming upsetting’, consisting of the subthemes ‘connections and acceptance over rules and expectations to fit in’ and ‘self-care as protective practice’; (6) ‘managing emotional responses during periods of upset’, consisting of the subthemes ‘affirmation by and “check-ins” from others’, ‘self-directed regulation: autonomy and action during distress’ and ‘letting it out: physical and emotional release’; and, finally, the theme (7) ‘leveraging own strengths – drawing strength from “within”’, i.e., using the strengths that individuals possess to be self-reliant and resilient^17^

### Topic modelling and cluster analysis plan

**Fig. 1:**
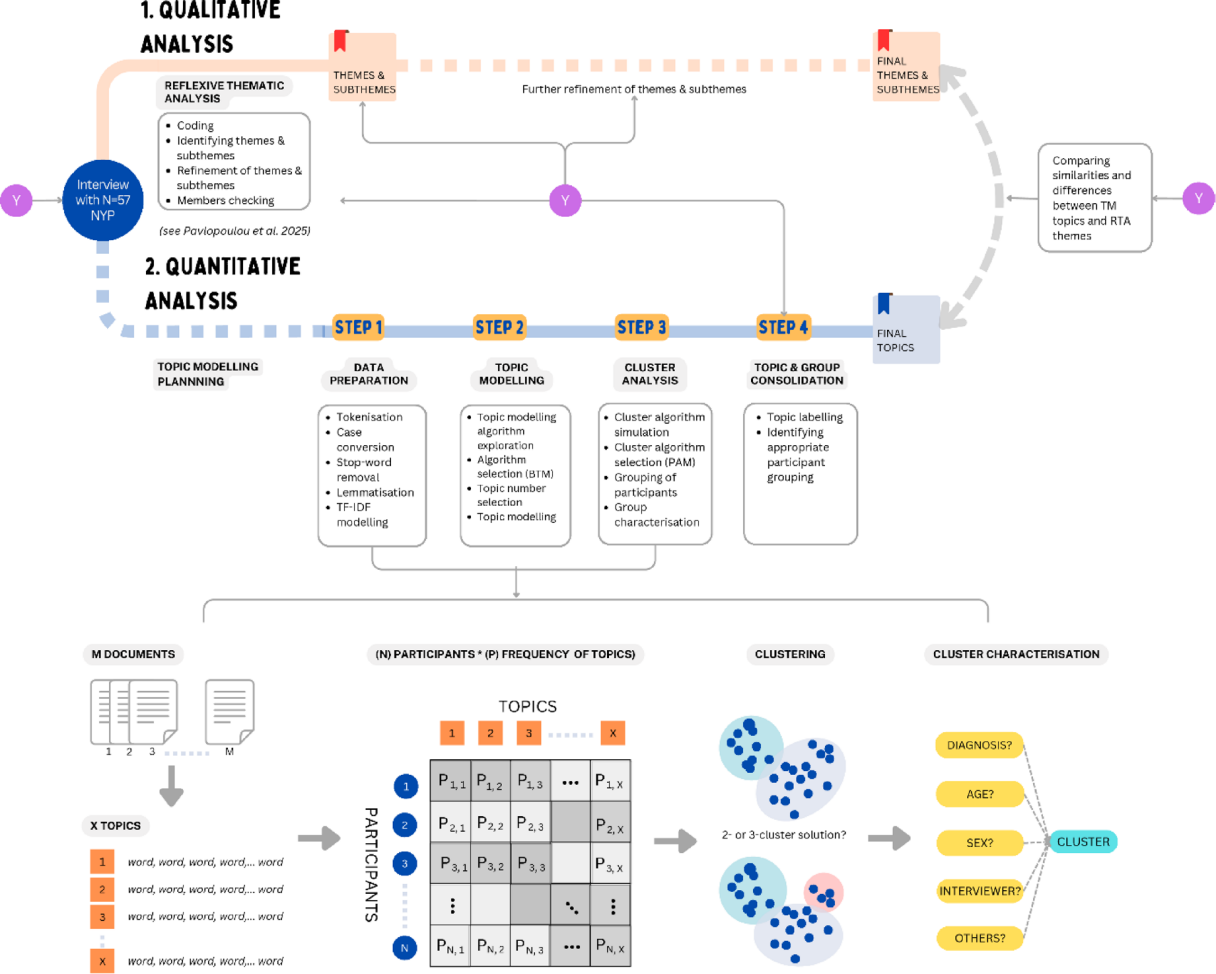
Schematic of the reflexive thematic analysis and topic modelling. Paths from interview data collection to the qualitative and quantitative topic modelling analyses for the *My Emotions and Me* study. The quantitative analysis commenced after the themes and subthemes of the qualitative analyses were identified. Only the quantitative analysis steps are presented in detail [qualitative analysis steps are described in 16]. The quantitative analyses Steps 1 and 2 were led by a quantitative researcher (LL) independently from the qualitative research. Inputs from neurodivergent young researchers (Y) were sought at different stages of the research including (1) to design the interview schedule and to refine research materials, (2) to label topics, (3) to compare the final topics and themes. Abbreviation: BTM = bi-term topic modelling algorithm; NYP = neurodivergent young participants, P = probabilistic occurrence of a topic, PAM = partitioning around medoids cluster algorithm, and TF-IDF = term frequency-inverse document frequency, Y = Youth researcher panel of RE-STAR.

TM commenced after preliminary RTA themes were generated to minimise the influence of the TM on our primary qualitative analysis. TM analysis consisted of four steps (Fig. 1). The first two steps were led by an independent computer scientist (LL). The remaining steps were led by the first author (SL), who participated as a coder in the earlier part of the RTA. Inputs were sought from Y-RP members at different stages (Fig. 1).

Step 1, data preparation, comprised several pre-processing stages applied across *documents*. Following Bastiaansen and colleagues^32^, a single document was defined as an interviewee’s response to a question or an utterance instead of the entire interview. We chose this approach to improve the stability and interpretability of the topic model despite the limited number of participants by TM standards. This approach is parallel to the qualitative theme generation, which can be based on a range of quotes from a participant instead of the entire interview.

The pre-processing stages were conducted using the Python package *Sklearn*^36^, and involved: (i) Tokenisation, i.e., dividing text from each interview response into a collection of single words (tokens); (ii) Case conversion, i.e., transforming all words into lower cases; (iii) Stop-word removal, i.e., removing common English words that were not useful for TM, (e.g., prepositions, numbers as identified using the Python *nltk* library), extended to include additional terms identified in the data: *“um”, “oh”, “okay”, “mhm”, “ah”, “uh”, “yes”, “get”, “yep”, “yeah”, “no”, “hm”, “wow”, “.”, “?”, “-”, “-”, “:”, “mm”,* and *“oop”*; (iv) Lemmatisation, i.e., generalising the text data by mapping all words to their base form (e.g., ‘walked’ or ‘walking’ becomes ‘walk’); and (v) Creation of a Term Frequency-Inverse Document Frequency (TF-IDF) model, in which words are assigned a frequency adjusted according to their capacity to exert influence in the document, based on both their frequency in an individual document and across all the documents^37^. Following previous approaches, we removed terms with less than 1% and more than 50% frequency in the documents [see e.g.,^38,39^].

In Step 2, TM was performed across all documents, i.e., responses to questions, blind to the RTA themes. The unsupervised Bi-term TM (BTM) was carried out using the Python package *bitermplus*^40^]. The BTM algorithm was selected instead of the traditionally used Latent Dirichlet Allocation (LDA). The BTM typically outperforms LDA when modelling short text in microblogging posts (e.g., tweets) with sparse word co-occurrence^40,41^, which are comparable in length to our young people’s verbal utterances/responses. BTM produced higher topic coherence from open-ended forms of such posts, including consumer surveys and disaster-related tweets, than the LDA^42,43^, with higher efficiency^41^ and accuracy^44^. Unlike the LDA, the BTM models the co-occurrence patterns of (unordered) word pairs instead of looking at single words, and across the whole set of documents, which is assumed to contain a mixture of topics, instead of focusing on separate documents. Each word pair or bi-term is assumed to be drawn from a specific topic, and the BTM can process overlapping and hierarchical topics, offering a greater flexibility to modelling topics in this context compared to the LDA^40,41^. The decision of how many topics were optimal per individual and across the dataset was informed by their perplexity and coherence scores, calculated by the *bitermplus* package^45,46^. The number of topics associated with the overall lowest perplexity while maintaining coherence was tested in 2 to 14 plausible numbers of topics.

In Step 3, we explored whether topics might be associated with participant clusters with meaningful characteristics such as their diagnosis. This was performed using R 4.1.1 on the RStudio 2023.12.1 using the package *Cluster*^47^. We first performed a cluster analysis to explore groups of individuals with similar distributions of topic frequencies or “topic profiles” (i.e., the counts of each topic divided by the number of counts of all topics across documents in a participant’s interview). The cluster analysis was performed blind to the participants’ identity. We used the *k*-medoids clustering or partitioning around the medoids (PAM) due to its robustness against outliers^48^, and since it produced replicable clusters when tested using simulated data with a well-defined group partition (Supplements).

Based on the literature demonstrating differences in the emotional expression and regulation difficulties in autistic young people and/or those with ADHD^7,16,49^, we explored a potential emergence of *k* = 2 or 3 clusters among the participants. The participant grouping into *k* = 2 clusters could, for instance, reflect distinctive emotional experiences of autistic adolescents or those with ADHD, where the autism + ADHD group has combined features of the two clusters. In contrast, *k* = 3 could reflect three distinct clusters of emotional experiences distinct to each diagnosis [see also^50,51^], although other predictors could also explain the clusters. Before the cluster analyses, topic frequency data were scaled by subtracting the mean and dividing by the standard deviation. They were subsequently transformed towards normality using the Yeo-Johnson transformation. We thus clustered the participants based on their scaled and normalised topic frequency data. The mapping of cluster memberships in the two- and three-cluster solution was explored using cross-tabulation. The degree to which clusters were well defined was assessed using the Silhouette and Caliński-Harbasz (CH) indices^52,53^. To evaluate the robustness of the clusters, we performed bootstrap resampling with 1000 iterations and computed both adjusted rand index (ARI) and normalised mutual information (NMI)^54,55^ for each resampled solution.

Following the cluster formation, we further explored whether cluster memberships were predicted by participant diagnosis or other potential explanatory variables such as age, sex, medication status, and the identity of the researcher interviewing the participant. This was performed using Chi-square analysis for categorical predictors, or logistic regression for continuous predictors. We first predicted cluster memberships using the predictors separately. Then, to examine the specificity of association between predictors and clusters, the significantly associated predictors with cluster memberships in the first stage were entered simultaneously in a single model to predict clusters in a second-stage analysis. Model fit was assessed using McFadden pseudo-R^2^^56^. To further characterise the clusters, we compared the frequencies of each topic across them twice, adjusting for the interviewer’s identity in the second comparison. In this explorative study, we did not correct for multiple comparisons across the topics^57^, but *post-hoc* comparisons among clusters were adjusted using Bonferroni correction. Since the association between clusters and diagnosis was confounded by the interviewers’ identity, we also conducted cross-tabulation and a chi-square test *post-hoc* for the association between these variables.

**Fig. 2:**
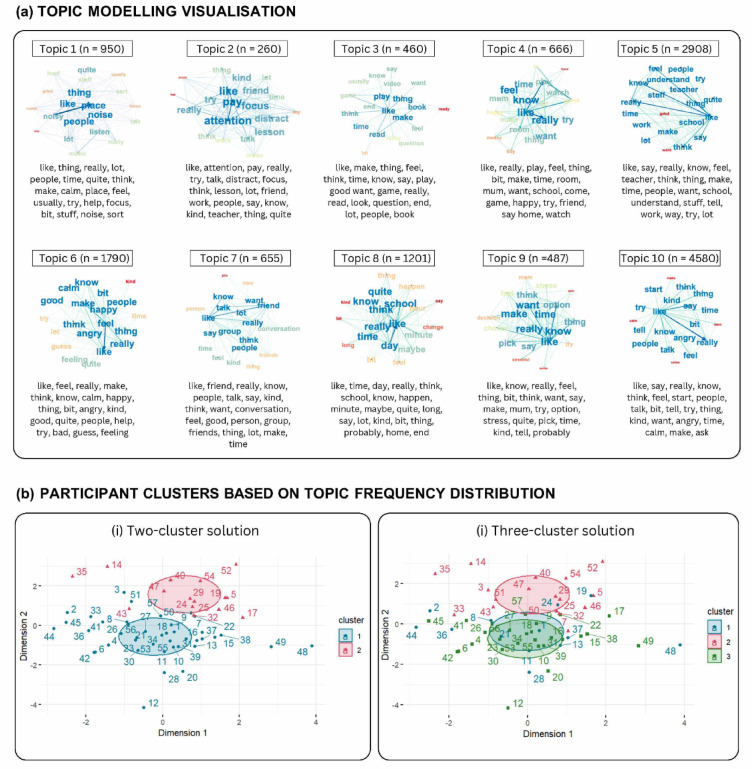
Topics and topic-based participant grouping. (**a**) Topics are displayed in word co-occurrence networks together with the top 20 representative words. The occurrences of these topics across documents are indicated in brackets. (**b**) Topic-based two- and three-cluster solutions of the participants partition around the medoid.

In Step 4, in line with the participatory research framework, academic researchers and Y-RP members collaborated as domain experts to label topics, thus incorporating the authentic voice and neurodivergent positionality in this process. The topic labelling involved collaborative examination and interpretation of topics in three 1-hour sessions. The first session focused on generating topic labels from the top 20 words and the word co-occurrence network representing each topic (Fig. 2). Labelling exercise continued to the second session, alongside discussions of the topics’ potential relations with the RTA preliminary themes. Topic labels were refined further by academic researchers through close reading of documents informing each topic^58^. In the third session, academic researchers and Y-RP members discussed the final labels and the interpretation of findings. Once the topic labels were decided, as an additional exploration, academic researchers examined the mapping between topics and themes to better understand their similarities and differences by assigning one RTA theme to the documents informing each topic. We then qualitatively inspected each topic distribution across themes, focusing on themes consisting of more than 14.3% of documents from each topic, the chance level for a document to be distributed across the seven themes.

## Results

### Topic modelling

The number of documents before any pre-processing was 17,953. After pre-processing 13,957 documents remained, as many short documents no longer had the retained words (e.g., one-word responses like “yes”).

The topic number = 10 resulted in the lowest perplexity score = 1 while maintaining coherence of -477.60 (Table S1). Thus, we chose to generate 10 topics during TM. The topics and their document counts (n) are shown in Fig. 2a, alongside images of the co-occurrence network, indicating most frequent words (i.e., larger font colour-coded blue), frequently co-occurring BTM word-pairs (i.e., bolder line connecting words), and top 20 representative words per topic.

### Topic frequency distribution-based clusters of participants

Figure 2b(i)–(ii) show the partitions of participants into two and three clusters based on the topic frequency. The two-cluster solution consisted of 42 (73.7%) and 15 (26.3%) participants in Clusters 1 and 2. The three-cluster solution consisted of 27 (47.4%), 17 (29.8%), and 13 (22.8%) participants in Clusters 1, 2, and 3, respectively (Table 2). Cross-tabulations indicated stability of Cluster 2 in both solutions (i.e., 12 [80%] of 15 members of Cluster 2 in the two-cluster solutions remained in the three-cluster solution). In the three-cluster solution, Cluster 3 appeared to be a subdivision of Cluster 1 of the two-cluster solution (n = 11 of 13, 84.6%), increasing the proportion of participants with ADHD in Cluster 1. The two-cluster, relative to the three-cluster solution, had a higher silhouette width (0.10 vs. 0.08) and a higher Calinski–Harabasz index (6.95 vs. 5.50). Although the silhouette widths suggested a modest cluster structure in both solutions, the two-cluster solution had better separation. Stability indices also showed comparably modest cluster stability for both solutions across resamples (two-cluster: ARI = 0.42, NMI = 0.41; three-cluster: ARI = 0.37, NMI = 0.44).

**Table 2:** Characteristics of the topic-based participant clusters.

Cluster predictors	(a) Two-cluster solution		Statistics		(b) Three-cluster solution			Statistics	
	Cluster 1 (n = 42)	Cluster 2 (n = 15)	*B/X*^2^	*p*	Cluster 1 (n = 27)	Cluster 2 (n = 17)	Cluster 3 (n = 13)	*B/X*^2^	*p*
Age, y (M, SD)	13.0 (1.43)	13.1 (1.22)	0.04	0.87	12.8 (1.41)	12.8 (1.25)	13.3 (1.43)	0.12	0.61
Sex (n, %)									
Male	27 (64.3)	11 (73.3)	0.41	0.76	17 (63.0)	13 (76.5)	8 (61.5)	1.06	0.66
Female	15 (35.7)	4 (26.7)			10 (37.0)	4 (25.5)	5 (38.5)		
Diagnosis (n, %)									
Autism	13 (31.0)	8 (53.3)	6.96	0.03*	7 (25.9)	9 (52.9)	5 (38.5)	10.1	0.037*
ADHD	22 (52.4)	2 (13.3)			16 (59.3)	2 (11.8)	6 (46.2)		
Autism + ADHD	7 (16.6)	5 (33.3)			4 (14.8)	6 (35.3)	2 (15.4)		
Ethnicity (n, %)									
Black	2 (4.8)	2 (13.3)	2.71	0.68	2 (7.4)	2 (11.8)	0 (0.0)	6.68	0.87
Asian	3 (7.1)	0 (0.0)			2 (7.4)	0 (0.0)	1 (7.7)		
Mixed	4 (9.5)	1 (6.7)			2 (7.4)	1 (5.9)	2 (15.4)		
White	32 (76.2)	12 (80.0)			20 (74.1)	14 (82.3)	10 (76.9)		
Others	1 (2.4)	0 (0.0)			1 (3.7)	0 (0.0)	0 (0.0)		
Free meal (n, %)									
Yes	6 (14.3)	3 (20.0)	0.27	0.70	4 (14.8)	4 (23.5)	1 (7.7)	1.42	0.51
No	36 (85.7)	12 (80.0)			23 (85.2)	13 (76.5)	12 (92.3)		
Interviewer (n, %)									
Interviewer 1 – MK	15 (35.7)	11 (73.3)	6.31	0.02*	11 (40.7)	10 (58.8)	5 (38.5)	1.72	0.45
Interviewer 2 – SL	27 (64.3)	4 (26.7)			16 (59.3)	7 (41.2)	8 (61.5)		
Stim Meds (n, %)									
Yes	15 (35.7)	5 (33.3)	0.03	> 0.99	12 (44.4)	7 (41.2)	1 (7.7)	5.60	0.07
No	27 (64.3)	10 (66.7)			15 (55.6)	10 (58.8)	12 (92.3)		

The association between the demographic predictors and interviewer identity and clusters in the (a) two-cluster and (b) three-cluster solutions. B-coefficient statistics are reported for analyses involving continuous variables (i.e. age), while X^2^ statistics are reported for the remaining. Cross-tabulations of counts and percentages of all levels of categorical predictors are presented for each cluster for ease of inference. Significance threshold *p < 0.05.

### Association between clusters and participant characteristics

#### Two-cluster solution

Diagnostic status was associated with cluster membership in the two-cluster solution (χ^2^[2, n = 57] = 6.96, *p* = 0.030), with selectively higher proportion of participants with ADHD in Cluster 1 relative to Cluster 2, compared with autistic (χ^2^ [1, n = 45] = 5.74, *p* = 0.022), or autistic + ADHD participants (χ^2^[1, n = 36] = 5.67, *p* = 0.028) (Table 2), which were more evenly distributed across the clusters. However, more participants were interviewed by SL in Cluster 1, and more were interviewed by MK in Cluster 2 (χ^2^ [1, n = 57] = 6.31, *p* = 0.020). Follow-up multinomial logistic regressions, controlling for interviewer identity, led to a non-significant association between diagnosis and clusters (*p* = 0.057). The model fit predicting the 2‐cluster solution using interviewer identity and diagnosis reached a moderate value (McFadden’s R2 = 0.17).

Lower frequencies of Topic 1 (*t* = 4.19; *p* < 0.001), Topic 3 (*t* = 4.19; *p* < 0.001), Topic 6 (*t* = 3.89; *p* < 0.001), and Topic 9 (*t* = 4.08; *p* < 0.001), and higher frequencies of Topic 4 (*t* = 2.37; *p* = 0.021), Topic 8 (*t* = 2.62; *p* = 0.011), and Topic 10 (*t* = 2.61; *p* = 0.011) were found across documents in Cluster 1 than Cluster 2 (Table 3a).

**Table 3:** Topic frequency comparison across participant clusters.

	(a) Two-cluster solution						(b) Three-cluster solution								
	Cluster 1 (n = 42)		Cluster 2 (n = 15)		Statistics		Cluster 1 (n = 27)		Cluster 2 (n = 17)		Cluster 3 (n = 13)		Statistics		Post-hoc
Topics	Mean	SD	Mean	SD	*t*	*p*	Mean	SD	Mean	SD	Mean	SD	*F*	*p*	
Topic 1	1.34	1.53	2.35	2.12	-1.99	0.052	1.00	0.96	1.84	2.21	2.54	1.96	4.03	0.023*	3 > 1*
Topic 2	1.71	1.39	2.36	1.35	-1.57	0.12	1.30	1.06	2.66	1.43	2.08	1.50	6.06	0.004**	2 > 1**
Topic 3	2.60	1.59	4.66	1.72	-4.19	< 0.001***	2.36	1.38	5.02	1.69	2.31	0.98	21.7	< 0.001***	2 > 1***; 2 > 3***
Topic 4	5.47	3.26	3.34	1.92	2.37	0.021*	6.17	3.51	4.08	2.29	3.37	2.02	5.09	0.009**	1 > 3*
Topic 5	22.5	6.60	21.6	5.13	0.48	0.63	23.0	6.19	20.2	5.63	23.4	6.80	1.39	0.26	–
Topic 6	11.8	4.14	17.0	5.18	-3.89	< 0.001***	11.4	4.01	16.9	5.35	12.1	3.63	8.82	< 0.001***	2 > 1***; 2 > 3*
Topic 7	4.95	3.88	4.53	2.34	0.40	0.69	5.36	3.79	5.04	3.73	3.52	2.39	1.24	0.30	–
Topic 8	10.2	3.63	7.55	2.14	2.62	0.011*	9.05	2.56	7.39	2.43	13.1	3.76	15.3	< 0.001***	3 > 1***; 3 > 2***
Topic 9	2.69	1.73	4.88	1.95	-4.08	< 0.001***	3.25	1.98	3.54	2.00	2.93	2.24	0.33	0.72	–
Topic 10	36.8	6.85	31.8	4.87	2.61	0.011*	37.2	6.62	33.4	5.57	34.7	7.83	1.84	0.17	–

Topic frequency mean and standard deviation (SD) across clusters. Significance thresholds *p < 0.05; **p < 0.01; ***p < 0.001

#### Three-cluster solution

Diagnosis was also associated with clusters in the three-cluster solution (χ^2^ [4, n = 57] = 10.1, *p* = 0.037), which was primarily influenced by diagnostic differences in Clusters 1 and 2 (χ^2^ [2, n = 44] = 9.77, *p* = 0.012), but not Clusters 1 and 3 (χ^2^ [2, n = 40] = 0.74, *p* = 0.80), or 2 and 3 (χ^2^ [2, n = 30] = 4.69, *p* = 0.11). Further examination by diagnostic groups pairwise, again reflected higher relative proportion of participants with ADHD in Cluster 1 than 2 compared to the autistic (χ^2^ [1, n = 28] = 7.53, *p* = 0.008), or autistic + ADHD participants (χ^2^ [1, n = 34] = 7.89, *p* = 0.011). The interviewers’ identity was not associated with the clusters. Non-medicated participants appeared higher, but not significantly, in Cluster 3 compared to other clusters (*p* = 0.07) (Table 2). The multinomial regression model predicting the three‐cluster solution by diagnosis and interviewer identity had a modest fit (McFadden’s R2 = 0.09).

Cluster effects on topic frequencies were found for Topic 1 (*F* = 4.03; *p* = 0.023) and Topic 4 (*F* = 5.09; *p* = 0.009), with higher frequencies across documents in Cluster 3 than in Cluster 1 for Topic 1 (*p* = 0.025), and in Cluster 1 than in Cluster 3 for Topic 4 (*p* = 0.018), respectively. Cluster effects were also found for Topic 2 (*F* = 6.06; *p* = 0.004), with higher topic frequencies in Cluster 2 than in Cluster 1 (*p* = 0.004); for Topics 3 (*F* = 21.7; *p* < 0.001) and 6 (*F* = 8.82; *p* < 0.001), with higher frequencies for both topics in Cluster 2 than in Clusters 1 (*p*s < 0.001), and in Cluster 3 (*p*_Topic3_ < 0.001; *p*_Topic6_ = 0.013), and finally for Topic 8 (*F* = 15.3; *p* < 0.001), with higher frequency across documents in Cluster 3 than Clusters 1 and 2 (*p*s < 0.001) (Table 3b). These findings remained when we adjusted for interviewer identity and additionally revealed a significantly higher frequency of Topic 4 across documents in Cluster 1 than in Cluster 2 (Table S2).

#### Additional post-hoc analyses

Diagnosis was significantly associated with interviewer identity (χ^2^ [2, n = 57] = 7.96, *p* = 0.020). Seventy-five percent of participants with ADHD were interviewed by SL, 66.7% of autistic participants were interviewed by MK, and an equal number of autistic + ADHD participants were interviewed by each researcher.

### Topics and themes

The ten topics were labelled: (1) ‘Sensory stimulation and experience’, (2) ‘Struggle to maintain focus and interest’, (3) ‘Engaging in learning and leisure activities’, (4) ‘After-school relaxation or obligation’, (5) ‘Stressful situations at school’, (6) ‘Experience and expressions of emotions’, (7) ‘Navigating around friendships and peer relations’, (8) ‘Time, changes, and emotional experience’, (9) ‘Struggle to decide under pressure’, and (10) ‘Managing annoyance, frustration and anger’. Example topic documents are in Table S3.

**Fig. 3:**
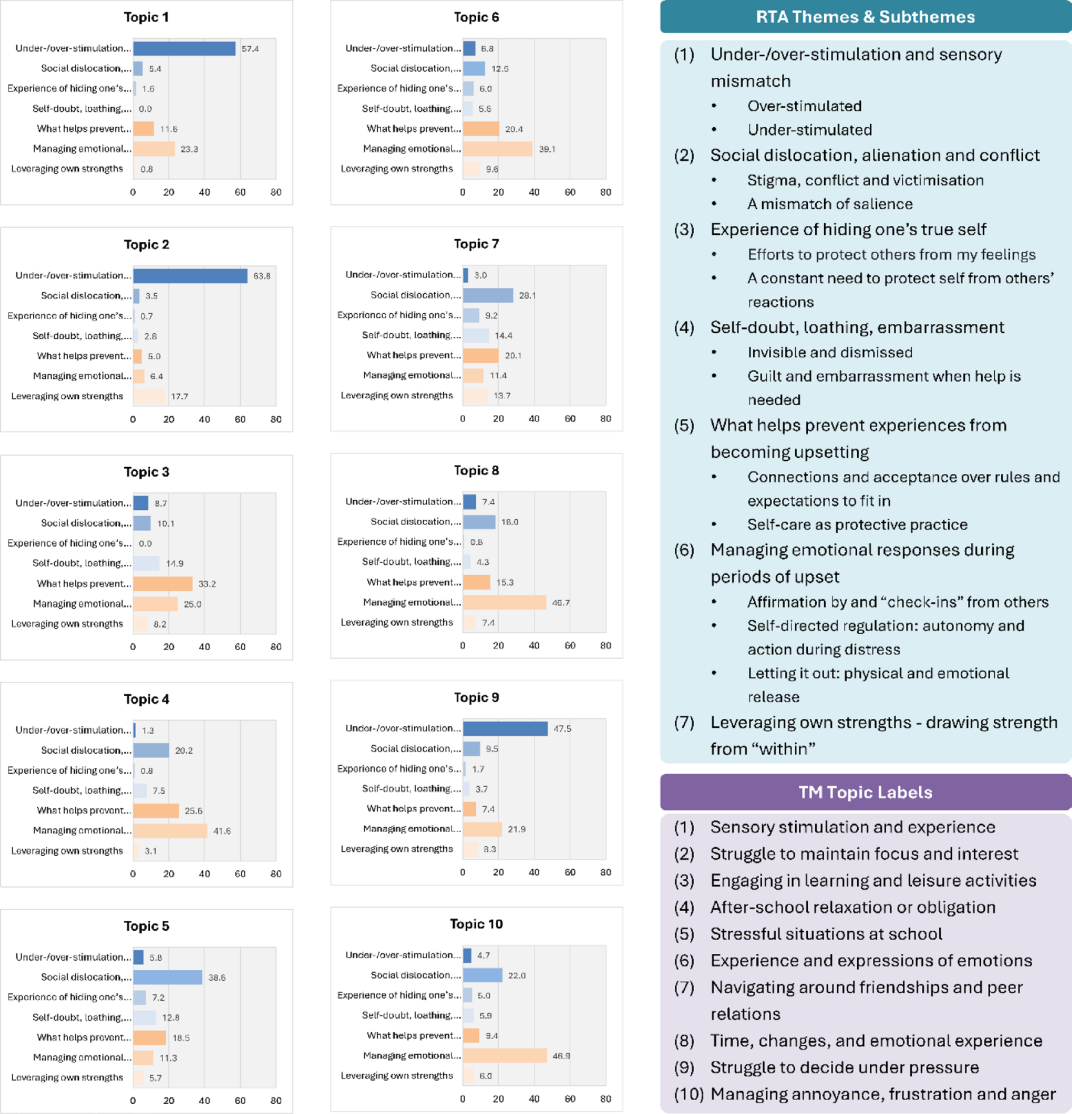
Representation of reflexive themes in the documents representing each topic. The ten TM topics and the proportions of documents expressing each reflexive theme within each topic. Abbreviation. TM = topic modelling.

TM topics and labels, RTA themes and subthemes, and the percentages of topic documents reflecting each theme are shown in Fig. 3. Within each topic, documents were related to at least one theme above the chance level of 14.3% among the documents. Topics 1, *‘sensory stimulation and experience’*; 2, *‘struggle to maintain focus and interest’*; and 9, *‘struggle to decide under pressure’* stood out in their relations with the theme *‘under-/over-stimulation and sensory mismatch’*. Furthermore, Topics 1 and 9, which described the feeling of being overwhelmed, aligned with the RTA subtheme *‘over-stimulated’* (Table S3a, i), while Topic 2, which was related to classroom boredom, aligned with the subtheme *‘under-stimulated’* (Table S3b). In addition, Topics 1, 2 and 9 were related to a few other themes, albeit to a much less extent: Topics 1 and 9 contained documents reflecting the theme *‘managing emotional responses during periods of upset’*, while Topic 2 contained some documents reflecting the theme *‘leveraging own strengths’*, reflecting conversations about hyper-focusing (Table 3a).

Both Topics 3, *‘engaging in learning and leisure activities’*, and 6, *‘experience and expressions of emotions’*, relate more strongly to *‘what helps prevent experiences from becoming upsetting’* and *‘managing emotional responses during periods of upset’* than other themes. Topic 3 documents appeared related to *‘what helps prevent experiences from becoming upsetting’* more strongly than *‘managing emotional responses during periods of upset’*—it also related to a lesser extent to *‘self-doubt, loathing and embarrassment’*, whereas Topic 6 documents showed a reverse pattern. Perhaps learning and leisure occurred more likely in the absence of upset, or to have a preventative role against upset, instead of as a way to manage emotions during upset. Contrastingly, emotional expressions were more likely experienced while managing upsets, and less so when they were prevented.

Topics 4, *‘after-school relaxation or obligation’*, and 5, *‘stressful situations at school’* differentiated between school and after-school settings (e.g., home) in relation to the young people’s emotional experience. Topic 4 was related strongly to *‘what helps prevent experiences from becoming upsetting’* and *‘managing emotional responses during periods of upset’*, suggesting a restorative/supportive role of after-school settings for the young people. To a lesser extent, Topic 4 was also related to *‘social dislocation, alienation, and conflict’,* reflecting the conflicts associated with parent-imposed obligations (Table S3d). Topic 5 was related primarily to *‘social dislocation, alienation, and conflict’*, aligned with the subthemes *‘stigma, conflict and victimisation’* and *‘a mismatch of salience’*; and to a much lesser extent with the theme *‘what helps prevent experiences from becoming upsetting’*, reflecting the predominantly hostile experiences at school (Table S3e).

Topic 7 *‘navigating around friendships and peer relations’* was related to *‘social dislocation, alienation, and conflict’, ‘self-doubt, loathing and embarrassment’,* and *‘what helps prevent experiences from becoming upsetting’,* denoting the ambivalent role of peers in young people’s lives – some were sources of upsets and self-doubts, while others helped prevent them.

Topics 8 *‘time, changes and emotional experience’* and 10 *‘managing annoyance, frustration, and anger’* were related primarily with *‘managing emotional responses during periods of upset’*. Topic 8 highlighted the dynamics of the experiences, such as their lengths and changes. Both topics also contained documents expressing *‘social dislocation, alienation, and conflict’*, reinforcing their proximity to upset. Topic 8, to a much lesser extent, was also related to *‘what helps prevent experiences from becoming upsetting’*.

The topic-theme mapping also revealed that themes *‘experience of hiding one’s true self’*, *self-doubt, loathing, and embarrassment*, and *‘leveraging own strengths’,* were consistently under-represented across topics.

## Discussion

We explored the similarities and differences of outputs from TM and RTA used for analysing transcripts of semi-structured interviews with 57 autistic adolescents and/or those with ADHD, considered a large sample for qualitative interviews^29,30^ although relatively small for TM. As a secondary data exploration, TM was performed independently from, and commenced after, the generation of reflexive themes that have been published elsewhere^16,17^. We identified ten TM topics, some closely mapped with, and others unrelated to RTA themes or subthemes. The topics appear associated with diagnostic status, related to a high proportion of participants with ADHD in one cluster, although this was potentially confounded by the study interviewers’ identity.

Topics identified through TM generally represent broad concepts comparable to the RTA themes. Some topics map well to the RTA themes or subthemes. For instance, the topics ‘sensory stimulation and experience’, ‘struggle to decide under pressure’, and ‘struggle to maintain focus and interest’ are part of the RTA theme ‘under-/over-stimulation and sensory mismatch’ and its subthemes. The topic ‘managing annoyance, frustration, and anger’ resembles the theme ‘managing responses during periods of upset’, and its subtheme ‘letting it out: physical and emotional release’ resembles the topic ‘experience and expressions of emotions.

These resemblances are interesting, especially since the chosen TM algorithms are ignorant of our research questions about everyday upsetting experiences and what helps manage them in neurodivergent young people. Indeed, topics like ‘after-school relaxation or obligation’, ‘navigating around friendships and peer relations’, or ‘managing annoyance, frustration and anger’, for example, include a mix of themes related to both questions, rather than being structured around each one separately. Such mixtures might relate to the reduced nuance of the TM topics compared to the RTA themes, which have been discussed previously^32,33^. This highlights the need for more sophisticated algorithms, or the inclusion of additional steps in our methods, to separate documents relevant to each research question before beginning the TM process.

Some TM topics explicitly reveal the role of settings and temporal dynamics in the emotional experiences of the young people. The topics ‘stressful situations at school’ and ‘after-school relaxation or obligations’ particularly illustrate a contrast between the predominantly negative experiences of neurodivergent young people at school and the more balanced challenges outside school, where supportive influences are more present. This positions school as a particularly challenging environment for neurodivergent young people, aligning with the frequently reported *school-related distress* among the student subpopulation^59–61^ and providing post facto support for the RE-STAR team’s focus on understanding the emotional burden of neurodivergent young people *at school*^18,62^. Our topics differ from the RTA themes that focus on the phenomenology of the experience but do not emphasise the distinct patterns of supportive and negative emotional experiences across settings, suggesting a potential complementarity of the two outputs.

The themes ‘experience of hiding one’s true self’, ‘self-doubt, loathing, and embarrassment’, and ‘leveraging own strengths’ bear no resemblance to any topics. The topic-theme map also shows relatively infrequent occurrences of these themes across topics. This could be because, first, terms underlying the themes were discussed less frequently during the interviews, and consequently, the topics were ‘drowned out’, and second, especially for the theme of hiding or masking, its expression might be more implicit and use more varied terms, of which the semantic connections are lost to the machine. In these instances, human positionality and/or interpretations — shaped by the literature^63–65^ and the lived experiences of the young people in this study — have surpassed our inadequately trained machine algorithms in capturing the full richness of the neurodivergent experience.

This was partially mitigated in our novel approach by incorporating neurodivergent positionality during topic labelling and results interpretation. Topic labels co-constructed by academic researchers and neurodivergent youth co-researchers from reduced data (e.g., word co-occurrence and top 20 representative words) and subsequent closed reading of the interview response documents are imbued by the reflexivity of the young people who are familiar with the context, therefore crucially, foregrounding the topic interpretation on the lived experience of the neurodivergent community.

Exploratory attempts to quantify relations between topics and clinical diagnoses yielded inconclusive but interesting findings. The two-cluster solution had better separation and offered more parsimony—although both solutions demonstrated similarly modest cluster structure with resampling. The two clusters were predicted, with moderate fit, by diagnosis but also confounded by interviewer identity and thus should not be over-interpreted. The confounding influence of interviewer identity arose because quantitative topic modelling was a secondary analysis, and the study was not originally designed for this purpose. Our *post-hoc* analyses showed that one researcher interviewed more participants with ADHD alone, while another significantly interviewed more autistic participants by coincidence. It should be noted, however, that controlling for interviewer identity led the diagnosis-cluster association to just miss the significance threshold, indicating that we were potentially underpowered to differentiate between the clinical groups. A future mixed-methods study testing the usefulness of TM should balance participant diagnoses across interviewers or randomise interviewer allocation and could consider increasing the sample size or including neurotypical students as an additional comparison group to aid contrast. The latter would also ensure that experiences from all students are captured.

Based on the emerging differences of the finally labelled topics from the themes generated in the previous RTA (e.g., some labelled TM topics have highlighted the distinct influences of school compared to home/community, and these were not explicitly present in the RTA themes or subthemes; see Fig. 3) and the inherently distinct processes leading to these, we suggest that TM and RTA exhibit potential complementarity, whereby each method can be used to elaborate on, enhance, illustrate, or clarify the results of the other^66,67^. How these outputs, or the processes leading to them, may influence each other and are integrated in a truly mixed-method study is beyond our scope, and remains to be formally tested, ideally within a convergent parallel design where data are analysed separately yet concurrently using these distinct approaches^68^. Indeed, our experience suggests that TM and RTA are best conducted separately, allowing each method to engage fully with its own paradigmatic and philosophical foundations. Within a research team employing a mixed-method approach, one group of researchers using RTA generates themes through iterative, reflexive, interpretive, and context-sensitive processes, while another group using TM derives topics through replicable computational procedures, which are later labelled and interpreted by humans. Importantly, although the processes leading to topics and themes differ across these methods, both theme generation and topic interpretation and labelling are ultimately human-led and rely on critical examinations of the researchers’ assumptions and positionality.

Integration or synthesis of findings may be considered once outputs from the respective analyses are finalised—although see other ways for integrating methods and/or outputs between qualitative and quantitative research^69–71^. This requires openness, curiosity, and an appreciation of methodological plurality^15^. Tensions between researchers using the two methods may arise during integration and should be acknowledged. For example, the above interview content variability across interviewers may be perceived by machine-learning approaches as noise, while it is considered a source of richness and diversity in qualitative research. Ideally, integrating knowledge from the two methods would leverage the distinct strengths of each^13,14^. Most importantly, insights derived from one method should not be used to “validate” or “verify” the findings of another. Instead, the emphasis should be on fostering dialogue among researchers, exploring the complementarity of findings, and producing new knowledge.

One strength of this study is its methodological innovation in modelling topics. This includes treating interview responses instead of the entire participant interview as a unit of analysis to enhance the stability and interpretability of topics, therefore bringing the method closer in practice to qualitative logic. The novel incorporation of neurodivergent voices during topic labelling and interpretation of findings infused reflexivity and neurodivergent positionality into topics, which could be further expanded. One major limitation affecting the cluster analysis in this study was the coincident association between interviewer identity and specific diagnoses, which could be addressed by balancing participant diagnoses across interviewers or randomising interviewer allocation in future mixed-model studies. Another potential limitation is that topic labelling and TM interpretation were conducted after the generation of RTA themes, to prevent TM findings from influencing the primary RTA processes. Thus, the topic labelling and interpretation might be influenced by the RTA, exaggerating the overlapping findings. Ultimately, our in-depth study of topics and their comparisons with the RTA themes was confined to a specific strand of NLP, i.e., topic modelling. Future comparisons and formal examination of the complementarity between thematic analysis involving more sophisticated strands of NLP such as generative artificial intelligence and large language models, which has been claimed to have the potential for supporting an NLP-augmented qualitative analyses^64,65^, would be useful.

Our study has provided an additional example in the sparse literature on the potential use of TM in analysing interview data. Based on the differences shown by the finally labelled topics from the themes generated in the previously published RTA, and the inherently distinct processes leading to TM topics and RTA themes, we have suggested a potential complementarity between the two methods. Our proposal on how this could be further investigated, incorporating the views of youth researchers as domain experts in TM, may be implemented using advanced algorithms and more sophisticated NLPs.

## Supplementary Information

Below is the link to the electronic supplementary material.

Supplementary Material 1

## Data Availability

Copies of the original thematic analysis codes, topic modelling outputs, and anonymised transcripts will be available from the corresponding author upon reasonable request.
