# Supplementary material for: Comparing qualitative thematic analysis and machine-based topic modelling in the analysis of autistic and ADHD young people’s accounts of emotions
Source: Sci Rep. 2026 Jan 28;16:4615. doi: 10.1038/s41598-025-34570-7 (PMC12868839; doi:10.1038/s41598-025-34570-7)
Supplement: Supplementary file 1 — Supplementary Material 1 [file 41598_2025_34570_MOESM1_ESM.docx]

**Supplementary Material to Lukito, Li et al. (2025). Comparing qualitative thematic analysis and machine-based topic modelling in the analysis of autistic and ADHD young people’s accounts of emotions**

**Table S1. Perplexity and Coherence Scores**

| Topic number | perplexity | coherence |
| --- | --- | --- |
| 2 | inf | -439.14 |
| 3 | 3.38E+260 | -451.70 |
| 4 | 3.87E+273 | -450.63 |
| 5 | 1.38E+11 | -462.50 |
| 6 | 1.06E+63 | -457.58 |
| 7 | 5.02E+100 | -467.54 |
| 8 | 3.20E+129 | -470.19 |
| 9 | 6.13E+149 | -476.57 |
| **10** | **1.00E+00** | **-477.60** |
| 11 | 2.91E+01 | -471.66 |
| 12 | 2.67E+01 | -479.64 |
| 13 | 2.46E+01 | -478.03 |
| 14 | 2.27E+01 | -480.50 |

.

## **Exploration of Cluster Algorithm Using Simulation Data**

Simulated data were created in R using the library *gtools*, from three numeric vectors, each consisting of 10 variables with well-defined topic distribution: x1=[0.4, 0.4, 0.05, 0.05, 0.05, 0.05, 0.0, 0.0, 0.0, 0.0], x2=[0.0, 0.0, 0.0, 0.0, 0.05, 0.05, 0.05, 0.05, 0.4, 0.4] and x3=[0.0, 0.0, 0.0, 0.1, 0.4, 0.4, 0.1, 0.0, 0.0, 0.0]. Simulated data were created by copying each vector 20, 40, 60, 80, 100, or 120 times, while adding Dirichlet noise into each column, therefore producing matrices with rows of 60, 120, 180, 240, 300, and 360 participant data to test the cluster algorithms to produce a 3-cluster solution. In the case of the 2-cluster solution, we only used vectors x1 and x2 to create the simulated data, therefore producing matrices with 40, 80, 120, 160, 200 and 240 participant data to test the cluster algorithms.

We considered two cluster algorithms: (1) unsupervised *k*-means clustering and (2) *k*-medoids clustering, also known as partitioning around medoids (PAM). Both required the researcher to pre-specify the number of clusters (*k*), which suits our research purpose of testing whether the grouping could be related to diagnosis status. The PAM algorithm was known for its robustness against outliers and non-normally distributed data (Jin & Han, 2010). For each cluster algorithm, data were partitioned three times, and the agreement between participant assignment to clusters with their actual cluster membership was computed using Fleiss’ Kappa plotted against sample size on Figure S1(a, b).

| Figure S1. Cluster Membership Assignment Agreement |
| --- |
| \|  \|  \| \| --- \| --- \| |
| Fleiss’ Kappa agreement comparisons between the partitioning around medoids (PAM) and the k-mean clustering for the 2- and 3-cluster solutions plotted with increasing sample.size. |

The PAM algorithm assigned cluster membership on simulation data with mean inter-rater agreement Fleiss’ κ = .98 (‘almost perfect agreement’; range = .95-1.00) for the 2-cluster solution and κ = .72 (‘substantial agreement’; range = .65-.86) for the 3-cluster solution. Using the same simulation data, k-means algorithm assigned cluster membership with a κ = .72 (‘substantial agreement’; range = .34-.95) for the 2-cluster solution and κ = .37 (‘fair agreement’; range = .17-.48) for the 3-cluster solution. The PAM algorithm was chosen for the subsequent cluster analysis of topics.

**Comparison of Topic Frequency Among Clusters**

Table S2 showed the comparison of topic frequencies across documents among clusters for the two- and three-cluster solution, adjusting for interviewer identity. In the two-cluster solution, lower topic frequencies were found across documents in Cluster 1 relative to Cluster 2 for Topic 3 (*F*=21.6; *p*<.001), Topic 6 (*F*=10.2; *p*=.002), and Topic 9 (*F*=14.6; *p*<.001), and higher topic frequencies in Cluster 1 relative to Cluster 2 for Topic 4 (*F*=8.22; *p*=.006), Topic 8 (*F*=6.16; *p*=.016), and Topic 10 (*F*=4.60; *p*=.036) (Table S2a).

In the three-cluster solution, while adjusting for interviewer identity, cluster effects on topic frequencies were found for Topic 1 (*F*=3.92; *p*=.026), with higher occurrence across documents in Cluster 3 than in Cluster 1 (*p*=.027); Topic 2 (*F*=5.67; *p*=.006), with higher topic frequencies across documents found in Cluster 2 relative to Cluster 1 (*p*=.005); Topic 3 (*F*=21.7; *p*<.001), with higher topic frequencies found in Cluster 2 compared to Cluster 1 (*p*=.004) and to Cluster 3 (*p*=.005); Topic 4 (*F*=5.58; *p*=.006), with higher topic frequencies in Cluster 1 than in Cluster 2 (*p*=.043) and in Cluster 3 (*p*=.015); for Topic 6 (*F*=7.86; *p*<.001), with higher topic frequencies in Cluster 2 than in Cluster 1 (*p*=.001) and in Cluster 3 (*p*=.018); and finally Topic 8 (*F*=14.8; *p*<.001), with higher topic frequency across documents in Cluster 3 than Clusters 1 and 2 (*p*s<.001) (Table S2b).

Table S2. Topic Frequency Comparison across Participant Clusters Adjusting for Interviewer Identity

|  | 1. **Two-Cluster Solution** | | | | | | **(b) Three-Cluster Solution** | | | | | | | | |
| --- | --- | --- | --- | --- | --- | --- | --- | --- | --- | --- | --- | --- | --- | --- | --- |
|  | Cluster 1 (n=42) | | Cluster 2 (n=15) | | Statistics | | Cluster 1 (n=27) | | Cluster 2 (n=17) | | Cluster 3 (n=13) | | Statistics | | Post-hoc |
| Topics | Mean | SD | Mean | SD | *F* | *p* | Mean | SD | Mean | SD | Mean | SD | *F* | *p* |  |
| Topic 1 | 1.34 | 1.53 | 2.35 | 2.12 | 3.97 | .051 | 1.00 | 0.96 | 1.84 | 2.21 | 2.54 | 1.96 | 3.92 | .026* | 3 > 1* |
| Topic 2 | 1.71 | 1.39 | 2.36 | 1.35 | 1.91 | .17 | 1.30 | 1.06 | 2.66 | 1.43 | 2.08 | 1.50 | 5.67 | .006** | 2 > 1** |
| Topic 3 | 2.60 | 1.59 | 4.66 | 1.72 | 21.6 | <.001*** | 2.36 | 1.38 | 5.02 | 1.69 | 2.31 | 0.98 | 23.2 | <.001*** | 2 > 1***; 2 > 3*** |
| Topic 4 | 5.47 | 3.26 | 3.34 | 1.92 | 8.22 | .006** | 6.17 | 3.51 | 4.08 | 2.29 | 3.37 | 2.02 | 5.58 | .006** | 1 > 2*; 1 > 3* |
| Topic 5 | 22.5 | 6.60 | 21.6 | 5.13 | 0.32 | .57 | 23.0 | 6.19 | 20.2 | 5.63 | 23.4 | 6.80 | 1.44 | .25 | -- |
| Topic 6 | 11.8 | 4.14 | 17.0 | 5.18 | 10.2 | .002** | 11.4 | 4.01 | 16.9 | 5.35 | 12.1 | 3.63 | 7.86 | <.001*** | 2 > 1***; 2 > 3* |
| Topic 7 | 4.95 | 3.88 | 4.53 | 2.34 | 0.25 | .88 | 5.36 | 3.79 | 5.04 | 3.73 | 3.52 | 2.39 | 1.29 | .28 | -- |
| Topic 8 | 10.2 | 3.63 | 7.55 | 2.14 | 6.16 | .016* | 9.05 | 2.56 | 7.39 | 2.43 | 13.1 | 3.76 | 14.8 | <.001*** | 3 > 1***; 3 > 2*** |
| Topic 9 | 2.69 | 1.73 | 4.88 | 1.95 | 14.6 | <.001*** | 3.25 | 1.98 | 3.54 | 2.00 | 2.93 | 2.24 | 0.25 | .78 | -- |
| Topic 10 | 36.8 | 6.85 | 31.8 | 4.87 | 4.60 | .036* | 37.2 | 6.62 | 33.4 | 5.57 | 34.7 | 7.83 | 1.44 | .25 | -- |

Topic frequency mean and standard deviation (SD) across clusters. Significance thresholds **p*<.05; ***p*<.01; ****p*<.001.

Table S3. TM Topics and RTA Theme(s)

| **Top 20 Words** | **Example Responses** | | **RTA Theme(s)** |
| --- | --- | --- | --- |
| 1. **Topic 1: Sensory experiences and stimulation** | | | |
| like, thing, really, lot, people, time, quite, think, make, calm, place, feel, usually, try, help, focus, bit, stuff, noise, sort | Being in noisy places yeah thats one of my biggest ones I dont like noise I use earpods when I know I am going to a noisy place to muffle the sound so it doesnt really affect me when I have my earpods in | | **Under-/over-stimulation or sensory mismatch** |
|  | This is the thing that like makes me get overwhelmed sort of thing. So I don't like really big crowds. I really don't like needles and stuff like that and like loud noises or when people are shouting. If I've got to like if I'm giving a big list of jobs to do and I can't remember them all I get quite overwhelmed because I want to remember all of them that's it. | |  |
|  | Yeah I was actually really scared because I am quite sensitive to a lot of noise actually because of my autism and all that. | |  |
|  | Being in a noisy area where there's a lot going on makes me feel overstimulated. It makes me feel scared. And I don't understand what I'm scared of. | |  |
|  | Oh so if I'd like already had like a boring day or something and then I just had to go to that noisy environment then it would be harder to cope or it'll be a bit more overwhelming. But if I'd had a better day then if I went to noisy a place then I might be able to cope better. | |  |
|  | I just sort of go to a calm place and calm myself down. | | **Managing emotional responses during periods of upset** |
|  | Yeah it just-- I like to listen to music to calm me down because it keeps me much calmer and in my like own space. | |  |
|  | Whenever I'm feeling stressed or worried I go as far as possible from what's making me feel worried as far as possible. Even if that means on the other side of school or the other side of the house I go as far as possible preferably to a quieter some place. I love nature you know. | |  |
|  | Um if I was in a place that there were lots of people and there also happened to be music or something on and I was trying to read something and then someone might also be trying to talk to me at the same time then that is very much flap and spin. | |  |
| 1. **Topic 2: Struggle to maintain focus and interest (n=260)** | | | |
| like, attention, pay, really, try, talk, distract, focus, think, lesson, lot, friend, work, people, say, know, kind, teacher, thing, quite | | Well, I understand because I need to be listening but I don't mean to be not paying attention to the work purposely I just sometimes just drift off without realising. | **Under-/over- stimulation or sensory mismatch** |
|  |  | I do get quite bored I dont really have any examples I do get bored easily I dont know how to explain it |  |
|  |  | Teachers probably don't notice me being that bored because I look like I'm getting on with the work. But I'm also really bored though |  |
|  |  | I mean sometimes I get in trouble for chatting or getting distracted and then -- or not paying attention but I'm not really sure. But most of the time I don't really do it. Sometimes I'm not even talking but I get in trouble for it. And sometimes I am paying attention they just didn't realise. But I mean sometimes I get distracted and sometimes like -- actually yeah |  |
|  |  | Yeah if I don't find them interesting then I won't pay attention in the lesson. |  |
|  |  | I guess if it's something I'm super into I just zone out of everything else. I only focus on that. | **Leveraging own strengths** |
|  |  | No no no. I literally like can do this and I'm not distracted. |  |
|  |  | They don't really notice. The way I usually get hyper-fixated is I think of a really cool concept. And I end up focusing on that so it looked like I was paying attention when really I'm not. |  |
| 1. **Topic 3: Engaging in learning and leisure activities** | | | |
| like, make, thing, feel, think, time, know, say, play, good want, game, really, read, look, question, end, lot, people, book | | Like if I was in school and it was a subject that I enjoyed then I'll be focused and I would just find it easier to get on with it if it was a subject that I like | **What helps prevent experiences from becoming upsetting** |
|  |  | Just to be enjoying what I'm doing just to be smiling or something. |  |
|  |  | The idea of that there was gonna be something at the end that rewards me. |  |
|  |  | Good music. Good music also boosts my mood. |  |
|  |  | Yeah a lot of the times because then I just forget I just watch the videos and forget that even happened. | **Managing emotional responses during periods of upset** |
|  |  | I usually just meditate and read to just like clear my mind. And don't overthink about it too much. So yeah. |  |
|  |  | There was a short-term method I used to put on music in the background but that only works if I know what I'm doing but I just don't enjoy it. If I'm learning something new I put music on the background I'm not learning anything because the music is taking more a hold of my mind than the new information is. But when I know something and it's just information that's coming up that's |  |
|  |  | If I would be able to go on my tablet I'd probably go and talk to my friends on like a call or play some video games it would take my mind off it. |  |
|  |  | It's just the fact that I know I need to get it done but my brain can't get me to do it. | **Self-doubt, loathing and embarrassment** |
|  |  | Yeah like just what's been said there when I do tests I try and go through them as quickly as possible and I end up getting something wrong because I hadn't read the question right. |  |
|  |  | Because I knew I could have done better. If I read it properly or I didn't impulsively do something. |  |
| 1. **Topic 4: After-school relaxation or obligation** | | | |
| like, really, play, feel, thing, bit, make, time, room, mum, want, school, come, game, happy, try, friend, say, home, watch | | Being messy I struggle to keep my room tidy because I my mum always makes me is like Do tidy your room tidy your room. And I mean obviously I want my room to look nice. And when I do tidy it feels nice. But it just seems to be get messy like a day after like straight away. And I just can't keep cleaning it it's just really and when I lose things because when you're being messy you lose things and it annoys me. I'm like trying to find something that I've lost and it gets me upset because I've lost. It just like really annoys me. But then I forget I've placed it somewhere. But because my because where I put it is messy like other things on top of it and it's just yeah it's quite hard. | **Social dislocation, alienation, and conflict** |
|  |  | Because I like my personal space and I've got a really small room so I feel like I shouldn't be pestered in there if that's where I go to calm down. Like and someone comes in and interrupts me. |  |
|  |  | It was a bit okay because I was very high tempered on those days because my mum thinks it was because I had my room had a lot of stuff [inaudible] all over it because I did -- because I didn't have a lot of space to be in my room. |  |
|  |  | I like I hold like use force on the door And push him out and I sit in front of the door like on the floor right in front of the door |  |
|  |  | Yeah I do have something nice I do have Youre Dead to Me which was the BBC podcast which is kind of comedic history lessons and then I have Dog Diaries stuff | **What helps prevent experiences from becoming upsetting** |
|  |  | Yeah this is not an actual video game this is a real life game. With laser tag guns so it's like a bit practically you're using technology but you're running. Exercise. |  |
|  |  | I get happy for a long period of time. And I love it because yesterday we went to a retro game shop and I love retro games. I love retro game consoles I love all the old stuff. So when we went into there I was really happy and I was happy for all the day. I was smiling and being happy all the day. |  |
|  |  | It's probably in the fact that it's not the end of the world. I can always watch it another time. It's not like the only time. With homework I'm a bit more like Okay have a deadline. If it's just watching TV or something it can be done any other time. |  |
|  |  | I often come and sit and speak to my mum about my worry. Yeah and I'll just watch TV and do something to take my mind off it. | **Managing emotional responses during periods of upset** |
|  |  | If I’m doing if I’m at home and I’m already quite stressed I like to either watch TV talk to my friends or play some games |  |
|  |  | I'll probably just watch TV on my laptop in my room or something because like not socialise with anybody because that would probably just make me the most annoying person. |  |
|  |  | I'll say It's under the bed don't worry don't worry. It's under the bed. Don't worry. And I watch TV and it'll calm me down for a bit. But maybe I'll jump out of my seat and look everywhere for it. And I'll find it I'll find it |  |
| 1. **Topic 5: Stressful situations at school** | | | |
| like, say, really, know, feel, teacher, think, thing, make, time, people, want, school, understand, stuff, tell, work, way, try, lot | | I think I have at some point but I don't really do that now so yeah. But I used to answer back at the teacher sometimes but then I thought it's not a nice thing to do so I don't do it anymore really. Unless I'm really really annoyed with them and I just say something small like That's not what we're meant to do I thought you said do this blah blah blah. But not something that just insults them. | **Social dislocation, alienation and conflict** |
|  |  | Yeah sometimes. I have to wear my blazer jump and polo shirt so I ask if I can take my blazer off but they say no and I start asking why and they say Because it's a rule. But then I can take off my jumper but I'm not allowed to take off my blazer because apparently it unites our school because the year seven eight and nine's wear a red jumper but the year 10s' and 11s' wear black. But everyone wears the same blazer so we have to keep it on. |  |
|  |  | Maybe it will be at school or something. I'll be drawing a really good picture -- not really good but I'll be doodling in my notebook. And then my maths teacher would be like You have to put that away now. I'll be quite annoyed because I was still listening. But I don't think some teachers understand I can listen as well as doing that. |  |
|  |  | It is normally when I'm -- because I can get stressed out you know stuff like that is happening in my friend group commotion around school commotion at home. When I do something wrong I get into trouble or something like that. I can get stressed over that. |  |
|  |  | Some teachers understand it because they might have had experience with people before who are like me and some teachers don't understand it. | **What helps prevent experiences from becoming upsetting** |
|  |  | They just listen. |  |
|  |  | But at home I feel like it's fine. I can do that because I don't think my parents don't judge. I'm not really sure. |  |
| 1. **Topic 6: Experience and expressions of emotions** | | | |
| like, feel, really, make, think, know, calm, happy, thing, bit, angry, kind, good, quite, people, help, try, bad, guess, feeling | | When I’m happy I tend to act more calm my body is more relaxed And I do tend to laugh a lot in my youth theatre sessions | **What helps prevent experiences from becoming upsetting** |
|  |  | Things that boost my mood like if I find out that I'm seeing my friends at the weekend or something. And then like we might be exciting or something. Then that could just make me a bit like make my mood a bit better. |  |
|  |  | Because I feel like I'm -- this flat -- I feel like I'm in a field of flowers or something when I get happy. I get really smiley I get really just -- I get love I get the feeling of love when I'm happy. |  |
|  |  | I probably like huff and puff quite a lot. | **Managing emotional responses during periods of upset** |
|  |  | Well especially I had this toy and I just got to the point where I was so angry that I broke that. But this one here it's actually meant to be broken. With the journal it's meant to be broken. It's not like I'm gonna make anyone upset by doing that. |  |
|  |  | I feel like that's my way of getting my emotions out is by howling. That's how I get my emotions out my nerves or being anxious by being surrounded by people |  |
|  |  | They could just try and comfort me and made me feel a bit better about it all. |  |
|  |  | She likes to give me hugs she doesn't hug a lot but when it comes to when I'm feeling upset she likes to give me hugs. And she likes to do everything she can because as I said she's got some of her own problems so she already finds it hard herself. But if I'm feeling upset she'll try and make me feel better. |  |
| 1. **Topic 7: Navigating around friendships and peer relations** | | | |
| like, friend, really, know, people, talk, say, kind, think, want, conversation, feel, good, person, group, friends, thing, lot, make, time | | Especially with my friends I feel like I'm ignored sometimes or a bit pushed out. I often feel left out a lot. Not really as much anymore but especially when I was younger I'd feel left out a lot. And when I'm with friends at cheer sometimes I feel a bit left out because they're all talking and I'm not really don't really fit in. Yeah that's about it really | **Social dislocation, alienation and conflict** |
|  |  | Yeah. When I'm joining a conversation about -- say my friends are talking about something I -- they talk about anime all the time and they don't realise I watch anime. They don't realise it 'cause -- I love anime but I don't watch it a lot. It will be like -- 'cause they'll start simplifying stuff. They'll go -- they'll be saying like blah blah blah blah blah blah. And then I'll join them but they'll be like -- I'll be like What were you guys talking about? and they'll be like Oh just an anime series. And I'm like Oh what's it called? It's fine you won't understand it. I |  |
|  |  | And I don't like being ignored. It's one of my -- and I also don't like it when I am talking and someone just starts a whole new conversation as if I wasn't even there. It's like Hello I'm here. It makes me feel like insignificant. It just makes me feel upset because it's like so they think they're more important. |  |
|  |  | I can express myself like normally with my best friends. But say if it's just another person I'd probably change myself because they wouldn't know who I really am. So they'd probably -- say if I'm excited they'd probably find me overexcited. If I'm like that like. | **What helps prevent experiences from becoming upsetting** |
|  |  | Just not speaking to them. Maybe not not speaking to them but remembering there are people who care about me respect me without speaking to people like my friends and that. Just remembering on my own that just calms me down saying I don't want to talk to this person if they don't want to treat me with respect why do I need them in my life in my circle of friends? |  |
|  |  | When I have something nice when they do something nice from the canteen. So like today they were selling ice lollies at school which they do on hot days. And I had the I think they're called twisters and they're really nice but I had it and I It made my day better. And I think having a really deep conversation with my friend and something happens in a subject which I find exciting and yeah. |  |
|  |  | I don't really distance myself but I don't talk to her as much as I used to when we first became friends last year. And I don't really think she's probably the friend for me because she's a bit more -- yeah. So when I go to college I'm going to hopefully make some new friends that are more like minded to me. |  |
|  |  | If they asked me I just think it would be awkward. Like I said at the beginning I just don't really like to talk about it how I'm feeling or sometimes I do if it's my friends my close friends. | **Self-doubt, loathing and embarrassment** |
|  |  | Not really. I'm still not that good at talking to new people. |  |
|  |  | It goes through my head they don't like me anymore. And they don't want me on the table. So I don't really have any other friends. We are a group of 10 on the table including me but not including it's nine. And I just don't really have any other friends. I don't want any other friends because there are friends that make me feel like I'm good enough. |  |
| 1. **Topic 8: Time, changes, and emotional experience** | | | |
| like, time, day, really, think, school, know, happen, minute, maybe, quite, long, say, lot, kind, bit, thing, probably, home, end | | At the same point because it builds up It does goes up quite quickly because I am finding myself more stuff more quickly | **Managing emotional responses during periods of upset** |
|  |  | If I was feeling irritable it would either last for like a while or it would only last for like half an hour. |  |
|  |  | Definitely finding something to do. Interacting with friends playing out going swimming with stuff like that. It's just mostly when I'm upset. So I'm not really doing nothing. Or say about school day or something like that. |  |
|  |  | I was just annoyed -- well it changed when she -- I've always know that she's not a nice person. She's always not been friendly so I've never really been in her way at all. But it changed fairly quickly because I just carried on with the thing. But it was more at that moment where I felt it. |  |
|  |  | I definitely dont know because its all in the heat of the moment really | **Social dislocation, alienation and conflict** |
|  |  | It's probably because it's not like this is the only time you can go on the laptop she's had all day. And as soon as I want to do it all of a sudden she wants to as well. |  |
|  |  | Yeah kind of. I'm quite good at getting homework done. But even if I have to stay late to do it I will get it done. It's just like the stress of having to get it done in time. |  |
|  |  | It lasts like once the lesson ends I'm not bored as much anymore. And like say if I've got maths which I'm quite bored in and then I've got engineering like straight after I'm quite -- I get a little bit excited actually. Because I've got a lesson that I really like afterwards. | **What helps prevent experiences from becoming upsetting** |
|  |  | It's definitely got less it's not happened as often now than it used to happen but there's still the occasional one but yeah. |  |
|  |  | No because the feelings are acquired as I always do my homework the last minute. And then I get negative house points if I don't get a high enough mark on my homework. |  |
| 1. **Topic 9: Struggle to decide under pressure** | | | |
| like, know, really, feel, thing, bit, think, want, say, make, mum, try, option, stress, quite, pick, time, kind, tell, probably | | I don't really know it's just having loads of options to choose -- it's a good thing but having too many -- 'cause you have to vary -- this is the best in that but this is also the best in that too. And you have to you can only pick one and it really annoys me because maybe there's a tie of which two are the best or something. | **Under-/over- stimulation or sensory mismatch** |
|  |  | I'm not good with making decisions at all. So sometimes my mum and my brother have a go at me because I'm trying to make a decision when I'll get into a state. So there was a time where there was two ways to walk. We could either go that way or the other way and I couldn't decide and I wanted them to choose but they said But this is like a challenge for you it's not hard just pick one way. And I didn't know which way to pick. And we were standing there for about 10 minutes and I was getting really frustrated because I couldn't pick which side. And I know it seems simple but because they both take you to the same place but I just couldn't pick. |  |
|  |  | I just like I don't know. It's all just like sometimes it can be quite scary for me but normally I am I think normally it all just resolves itself pretty much I don't know. I think like we're just I'm not like I don't know. I'm sorry. I'm not trying to I'm not being very helpful here but it's when there's too many options it can sometimes be a bit difficult but normally it gets resolved in the end and we find a solution that I like and you know and I think that I didn't have the you know we find ways to deal with it. |  |
|  |  | Not really. I kind of just like maybe a few like sighs of frustration I don't really do anything. And then I kind of just so afterwards after I get through it I kind of just like oh well and go back to what I was doing. | **Managing emotional responses during periods of upset** |
|  |  | I think try and breathe slower. Or like just go through the options again but sort of evaluate them more. I think it just happens less generally because now because from experience now when I go to a restaurant I've already been to I kind of know what I'm after a bit more. Normally when I go to a restaurant my parents are there so. |  |
|  |  | I don't know afterwards when it's happened to me with my mum she's apologised for pressuring me. Also sometimes I guess feels a bit expectant for me to make a decision. I don't know other people so I don't really know. |  |
| 1. **Topic 10: Managing annoyance, frustration and anger** | | | |
| Like, say, really, know, think, feel, start, people, talk, bit, tell, try, thing, kind, want, angry, time, calm, make ask | | Like he was trying to annoy me about last week and what I did was I just went outside and started playing like with my balls and then I just ignored him. And then he knew that I was just ignoring him so he just gave up. | **Social dislocation, alienation and conflict** |
|  |  | If I got quite angry it's probably because someone was annoying me and like they weren't listening. Yeah. They would just be like -- they would just not be listening if I was telling them something like important or something and they weren't listening and I had asked them to listen and it was important I told them it was important and they just didn't listen and I would get kind of angry at them and mad at them. |  |
|  |  | Them two are two different ones. So the first one says When my little brother comes in my room without asking. So it means like yesterday basically my brother came in my room like this is just for example my brother came in my room and he didn't ask. He just came in and started like keep on putting his foot in and out. So it was halfway in my room so he kept on saying I'm not in your room when he was in my room. So then I started screaming and everything and he just gets me really agitated. |  |
|  |  | Well I might if it's really bad and I thought that they were a close friend and stuff then yeah I might be quite upset at home and talking about it with my parents and stuff or yeah or something like that. | **Managing emotional responses during periods of upset** |
|  |  | Just take some deep breaths and just wait for it to turn up or just keep looking try my very best not to get annoyed and just stay calm. |  |
|  |  | If I dont cry I tend to feel it to like next period or what not like an hour or so but if I do cry too often that I will go to learning support just to get my anger out and I tend to do the work up there instead of |  |
|  |  | That I might need a minute to just go out by myself. Like without anyone speaking to me or people annoying me. Just to have a minute or something to fidget with to try and get my mind off it. Or to colour in or not sitting near the people that annoy me as well. |  |
